# Mutant Ataxin-2 Expression in Aged Animals Aggravates Neuropathological Features Associated with Spinocerebellar Ataxia Type 2

**DOI:** 10.3390/ijms231911896

**Published:** 2022-10-07

**Authors:** Inês T. Afonso, Patrícia Lima, André Conceição, Carlos A. Matos, Clévio Nóbrega

**Affiliations:** 1 Algarve Biomedical Center–Research Institute, 8005-139 Faro, Portugal; 2 Faculdade de Medicina e Ciências Biomédicas, Universidade do Algarve, 8005-139 Faro, Portugal; 3 Programa de Doutoramento em Ciências Biomédicas, Faculdade de Medicina e Ciências Biomédicas, Universidade do Algarve, 8005-139 Faro, Portugal; 4 Champalimaud Research Program, Champalimaud Center for the Unknown, 1400-038 Lisbon, Portugal

**Keywords:** spinocerebellar ataxia type 2, aging, polyglutamine diseases, autophagy, neurodegeneration

## Abstract

Spinocerebellar ataxia type 2 (SCA2) is a rare autosomal, dominantly inherited disease, in which the affected individuals have a disease onset around their third life decade. The molecular mechanisms underlying SCA2 are not yet completely understood, for which we hypothesize that aging plays a role in SCA2 molecular pathogenesis. In this study, we performed a striatal injection of mutant ataxin-2 mediated by lentiviral vectors, in young and aged animals. Twelve weeks post-injection, we analyzed the striatum for SCA2 neuropathological features and specific aging hallmarks. Our results show that aged animals had a higher number of mutant ataxin-2 aggregates and more neuronal marker loss, compared to young animals. Apoptosis markers, cleaved caspase-3, and cresyl violet staining also indicated increased neuronal death in the aged animal group. Additionally, mRNA levels of microtubule-associated protein 1 light-chain 3B (LC3) and sequestosome-1 (SQSTM1/p62) were altered in the aged animal group, suggesting autophagic pathway dysfunction. This work provides evidence that aged animals injected with expanded ataxin-2 had aggravated SCA2 disease phenotype, suggesting that aging plays an important role in SCA2 disease onset and disease progression.

## 1. Introduction

Spinocerebellar ataxia type 2 (SCA2) belongs to the group of polyglutamine (PolyQ) disorders, which are caused by an abnormal expansion of the CAG triplet within the coding region of the causative genes. SCA2 is the second most common type of spinocerebellar ataxia worldwide. It is a rare autosomal, dominantly inherited disease, with a higher prevalence among the Cuban population [1,2]. SCA2 is caused by a repeat expansion mutation in exon 10 of the ataxin-2 gene (*ATXN2*), producing an ataxin-2 protein with an abnormally long glutamine tract. The size of a normal *ATXN2* allele varies between 15 and 24 CAG repetitions [3,4], while expansions higher than 32 result in SCA2 [5]. The most common symptoms in SCA2 patients are motor ataxia and oculomotor dysfunction, with the slowing of saccades being a unique feature in SCA2 [6]. There is no cure for this disease [7].

Ataxin-2 is a protein ubiquitously expressed throughout the body and is known to be fundamental in early embryonic development [8]. It is an RNA-binding protein, with several functions, including stress granules’ formation and P-body structures [9,10,11,12], RNA regulators [13,14], lipid metabolism [15], calcium homeostasis [16,17], and lastly, in synaptic vesicle endocytosis [18,19,20]. Expanded ataxin-2 undergoes abnormal conformation, gaining a higher propensity to aggregate, and being predominantly localized in the cells’ cytoplasm [21]. SCA2 patients display aggregates and neurodegeneration in specific regions of the brain, which are two main disease hallmarks. These especially affect Purkinje cells, leading to cerebellar dysfunction and ataxic movements.

Aging is a complex process caused by the build-up of cellular damages over time, which ultimately leads to fertility and survival decline [22,23]. Since the brain is primarily composed of postmitotic cells, it is believed to be more vulnerable to aging effects [24]. Some of the aged brain hallmarks are protein, lipids, RNA and DNA intracellular inclusions, dysfunctional cellular clearance mechanisms (e.g., autophagic pathway), dysfunctional inter- and intra-cellular signaling, and neuroinflammation, which interestingly overlap with some of the SCA2 pathogenesis molecular mechanisms [25,26,27]. Moreover, post-mortem samples derived from aged individuals with no associated neurodegenerative disorder show abnormal protein accumulation [28] and brain shrinkage [29,30] as indicators of neurodegeneration, which happen in several neurodegenerative diseases, including SCA2.

SCA2 age of onset is around 36 years old [31], however it varies between individuals with the same number of repetitions, suggesting additional players in SCA2 pathogenesis. In this line, the question of how aging impacts SCA2 disease remains unknown. On one hand, the accumulation of expanded ataxin-2 over time could induce irreversible cellular damages, which leads to the onset of the symptoms and the progression of the disease. On the other hand, neuronal cells are affected by biological aging, which could decisively contribute to disease onset and/or progression. In this work, taking advantage of a recently developed SCA2 lentiviral mouse model [32], we injected lentiviral vectors encoding for mutant ataxin-2 in 3-month-old (3 m) and in 18-month-old (18 m) mice. We aimed at investigating the impact of aging on SCA2 neuropathological hallmarks associated with the expression of mutant ataxin-2. We found that older animals have more mutant ataxin-2 aggregates and a higher loss of neuronal markers, as compared with younger animals. This study opens a new avenue for understanding the impact of aging on the onset and progression of neurodegenerative diseases.

## 2. Results

### 2.1. Aged Animals Display More Aggregates and Neurodegeneration Hallmarks upon Expression of Mutant Ataxin-2

Aging is the primary risk factor for neurodegenerative diseases’ development, including Huntington’s disease (HD) [24,33], which like SCA2, belongs to the PolyQ disorders group. In these diseases, the number of CAG repetitions dictates the severity and disease onset. However, the age of onset is explained by the size of the CAG expansion in only 60% to 80% of cases, suggesting that other mechanisms may be responsible for modulating the moment when the disease emerges. In this line, we hypothesized that mutant ataxin-2 (MUTAtxn2) expression in aged animals aggravates SCA2 neuropathological hallmarks.

**Figure 1:**
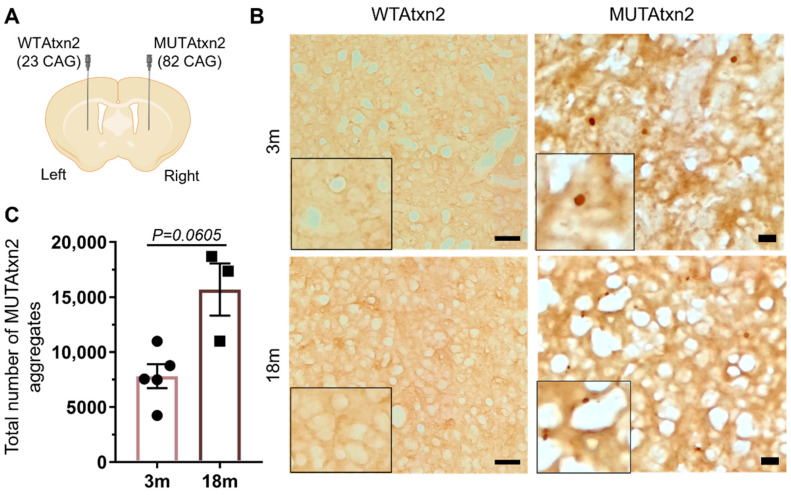
Aged animals displayed a higher number of MUTAtxn2 aggregates. (**A**) Schematic representation of striatal injection of lentiviral vectors encoding for WTAtxn2 (23 CAG repetitions) or expanded MUTAtxn2 (82 CAG repetitions). (**B**) Immunohistochemistry of mouse striatal slices stained for ataxin-2. Scale bar 20 µm. (**C**) Quantitative analysis of the number of aggregates 12 weeks post-MUTAtxn2 injection in the 3-month-old (*n* = 5) and 18-month-old animal groups (*n* = 3). Values are expressed ± SEM (standard error of the mean) (unpaired *t*-test with Welch’s correction).

To study the impact of aging on SCA2 pathology, we used a recently developed striatal SCA2 lentiviral mouse model [32]. Briefly, we injected wild-type ataxin-2 (WTAtxn2) in the left hemisphere of the striatum and MUTAtxn2 in the contralateral hemisphere, of 3-month-old (3 m) and 18-month-old (18 m) C57BL/6 mice (Figure 1A). Twelve weeks post-injection, we sacrificed the animals and evaluated the number of mutant ataxin-2 aggregates. Immunohistochemistry analysis for ataxin-2 revealed that the 18 m animal group displayed a higher number of aggregates in comparison to the 3 m animals (Figure 1B), 15,685 ± 2378 (*n* = 3) and 7808 ± 1094 (*n* = 5), respectively (Figure 1C), with a *p*-value of 0.0605.

**Figure 2:**
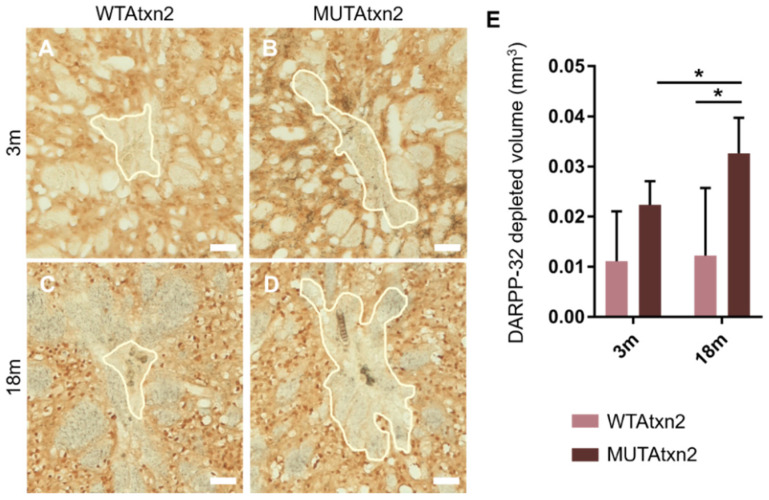
The hemisphere injected with MUTAtxn2 displayed a significantly higher volume of neuronal marker loss, compared to the hemisphere injected with WTAtxn2. (**A**–**D**) Representative immunohistochemistry of striatal sections stained with the DARPP-32 marker. Regions contained inside the white line have a depleted DARPP-32 signal. Scale bar: 50 µm. (**E**) Quantitative analysis of the DARPP-32 marker depleted volume in 3-month-old (*n* = 6) and 18-month-old (*n* = 3) animal groups. Values are expressed ± SEM (standard error of the mean) (one-way ANOVA, * *p* < 0.05).

To further evaluate the impact of MUTAtxn2 expression between the 3 m and 18 m animal groups, we immunostained brain sections for the neuronal marker, DARPP-32 (Figure 2A–E). The regions with depleted marker were measured and the total volume of neuronal marker loss was calculated. The quantification revealed a significant difference of DARPP-32 marker depletion between groups and within the aged animal group. No difference was found in the DARPP-32 loss when compared to the hemisphere injected with WTAtxn2, between young and aged animals (Figure 2A,C,E). On the contrary, in the hemisphere expressing MutAtxn2, there was a significant increase in neuronal loss in the aged animals (18 months), compared with the young group (3 months) (Figure 2B,D,E), *p*-value = 0.0464. In the same line, we found a significant difference in the neuronal loss volume when comparing the expression of WTAtxn2 and MUTAtxn2 in the aged animals, *p*-value = 0.0325.

**Figure 3:**
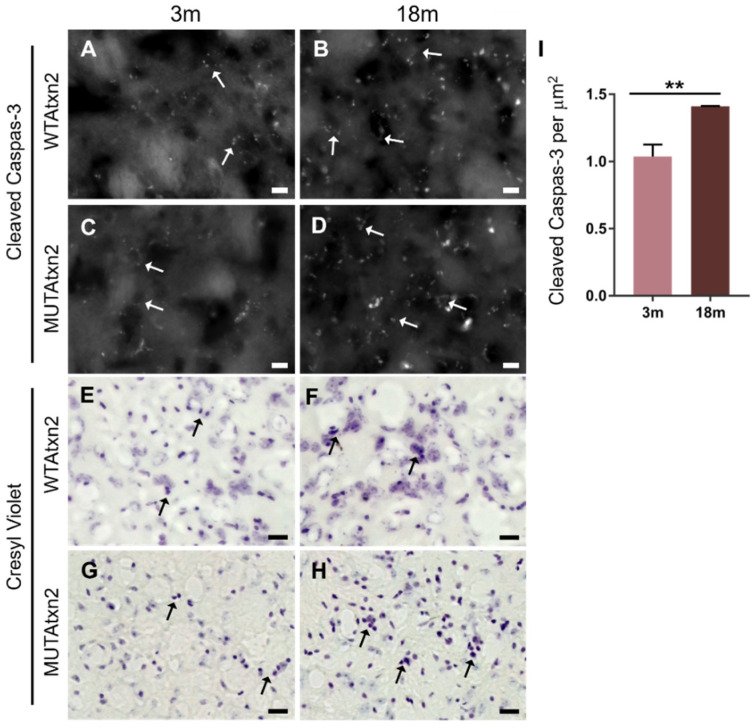
Cell death markers are increased in older animals injected with MUTAtxn2. (**A**–**D**) Representative images of striatal sections stained for cleaved caspase-3 (white arrows). Scale bar: 10 µm. (**I**) Quantitative analysis of cleaved caspase-3 marker in 3 m and 18 m animals injected with MUTAtxn2. (**E**–**H**) Representative cresyl violet staining of striatal sections in the 3 m and 18 m animal groups (black arrows). Scale bar: 20 µm. Values are expressed ± SEM (standard error of the mean) (unpaired *t*-test with Welch’s correction, ** *p* < 0.001).

We next performed immunostaining for cleaved caspase-3 as an indicator of cell death. The immunostaining detected a high signal of cleaved caspase-3 in the 18 m animal group injected with MUTAtxn2 (Figure 3B,D). We also found some cleaved caspase-3-positive cells in the hemisphere injected with WTAtxn2 (Figure 3A,B). Therefore, when quantifying the cleaved caspase-3 foci, we normalized the values obtained for the basal data found in the WTAtxn2-injected hemisphere. We found that in aged animals, there was an increase in the number of cleaved caspase-3-positive cells, compared to young animals (*p* = 0.0083). Furthermore, we performed cresyl violet staining, in which we observed an increased presence of pycnotic nuclei in the 18 m animals group injected with MUTAtxn2, in comparison with the 3 m injected animals (Figure 3C,D). 

### 2.2. Aged Animals Displayed More Astrogliosis upon Injection of Mutant Ataxin-2

**Figure 4:**
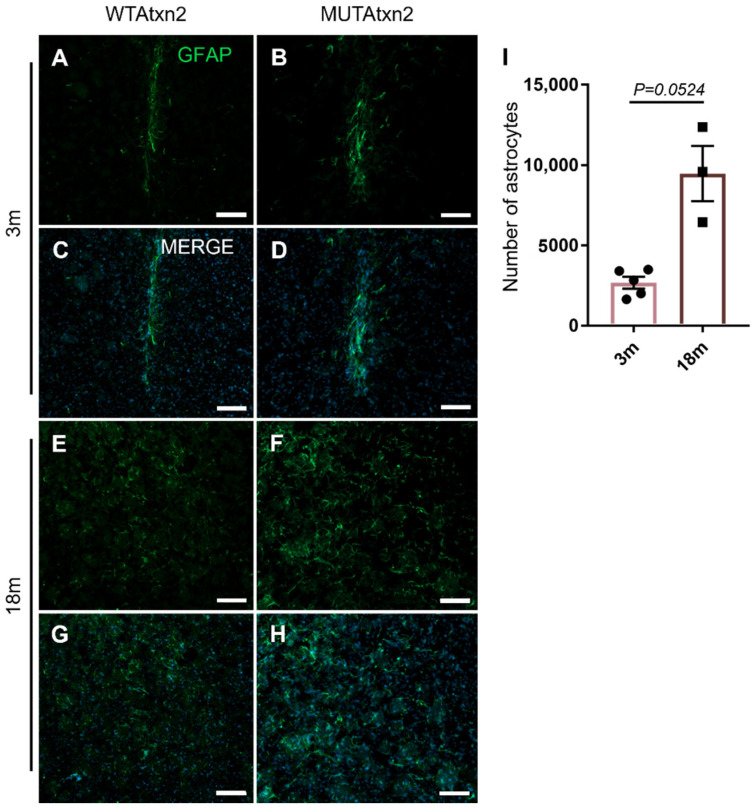
Aged animals displayed a significantly higher number of activated astrocytes. (**A**–**H**) Representative immunohistochemistry of striatal sections stained with GFAP. Green: astrocytes; blue: DAPI. Scale bar: 500 µm. (**I**) Quantitative analysis of the number of astrocytes in the 3 m (*n* = 5) and 18 m (*n* = 3) animal groups. Values are expressed ± SEM (standard error of the mean) (unpaired *t*-test with Welch’s correction).

Astrogliosis is a process where astrocytes change, becoming more reactive and present in larger number, often being associated with an insult or diseased tissue [34]. In the SCA2 striatal lentiviral mouse model, we showed that MUTAtxn2 expression induces astrogliosis [32]. Therefore, we hypothesized that aging could exacerbate astrogliosis. For that, we next stained striatal sections of the animals injected with the GFAP marker (Figure 4A–H). We found that in the MUTAtxn2-expressing hemisphere, there was a higher GFAP immunoreactivity than in the WTAtxn2 hemisphere, both in the young and in the older animal groups. In fact, we found that the 18 m animal group had a significantly higher number of astrocytes, in comparison with the 3 m animal group, 9467 ± 1715 (*n* = 3) and 2672 ± 368.7 (*n* = 5) astrocytes, respectively (Figure 4I).

### 2.3. Specific Aging Hallmarks Are Altered upon MUTAtxn2 Expression

López-Otín and colleagues described nine cellular and molecular hallmarks that contribute to the aging process [25]. Thus, we next aimed at studying some of those hallmarks in the experimental groups, specifically loss of proteostasis, mitochondrial dysfunction, and altered intercellular/interneuron communication. For that, we extracted RNA from striatal punches of the hemisphere injected with WTAtxn2 and MUTAtxn2 and performed RT-qPCR assays for different aging hallmarks.

**Figure 5:**
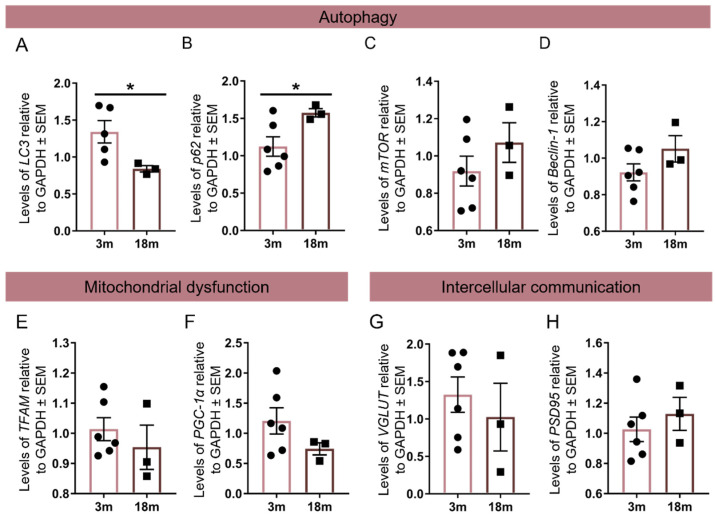
Assessment of aging hallmarks’ alteration upon MUTAtxn2 expression. mRNA relative levels in the striatum of SCA2 lentiviral mouse model, of 3 m (*n* = 5–6) and 18 m (*n* = 3) animals, twelve weeks post-injection. (**A**–**D**) Autophagy markers LC3, p62, mTOR, and beclin-1 were evaluated. LC3 levels were significantly decreased in the 18 m group compared with the 3 m group. (**E**,**F**) Mitochondrial dysfunctional markers evaluated were TFAM and PGC-1α. (**G**,**H**) Inter-neuronal communication markers VGLUT and PSD95 were evaluated in the 3 m and 18 m animal groups. No difference was found in these markers. Values are expressed ± SEM (standard error of the mean) (unpaired *t*-test with Welch’s correction, * *p* < 0.05).

We previously showed that the autophagy pathway is impaired in SCA2 and other polyglutamine diseases, and that it can be targeted as therapy [32,35,36,37]. Therefore, we analyzed the mRNA levels of key autophagic genes in the animals injected with MUTAtxn2, namely LC3, mammalian target of rapamycin (mTOR), p62, and beclin-1. Our results suggest alterations in LC3 and p62 levels between the experimental groups with a statistical significance of 0.0274 and 0.0172, respectively (Figure 5A,B). LC3 fold-change in the MUTAtxn2-injected groups was 1.342 ± 0.1515 (*n* = 5) for the 3 m animal group, and 0.8404 ± 0.04288 (*n* = 3) for the 18 m animal group (Figure 5A). The p62 fold-change levels in the MUTAtxn2-injected groups were 1.123 ± 0.1302 (*n* = 6) for the 3 m animals and 1.574 ± 0.05535 (*n* = 3) for the 18 m animal group (Figure 5B). No significant difference was found between the experimental groups in the mTOR and beclin-1 markers (Figure 5C,D).

Alterations in mitochondrial function were evaluated by analyzing alterations of expression in TFAM and PGC-1α genes. No significant alterations were found between the 3 m and 18 m groups for any of these markers (Figure 5F,G).

Finally, to evaluate inter-neuronal communication, we analyzed VGLUT and PSD95 levels. Our results did not show any significant difference between the 3 m and 18 m groups for any of these genes (Figure 5H,I).

## 3. Discussion

While the aging process has been widely associated with neurodegenerative disorders, few studies have demonstrated direct a link between aging and PolyQ diseases [33,38], and no study reported its impact on SCA2. The molecular mechanisms triggering mutant protein aggregation and neurodegeneration in SCA2 remains unknown, so we proposed that alteration in age-regulated processes may play a decisive role in SCA2 disease onset. This hypothesis is based on studies that show that protein aggregates accelerate age-related processes [38,39,40]. High insoluble proteins from aged *C. elegans* and mouse brains were able to induce amyloid-β aggregation in vitro [40]. Furthermore, ataxin-3 transient expression in early and late *Drosophila* stages showed that polyglutamine aggregates have distinct features [38]. They propose that aggregates from later *Drosophila* stages had higher toxicity and contribute to a later disease onset.

In this line, we found that lentiviral expression of mutant ataxin-2 in aged C57BL/6 mice significantly increases SCA2 neuropathological hallmarks, demonstrated by a high number of mutant ataxin-2 aggregates and neurodegeneration features (e.g., loss of DARPP-32 neuronal marker), as compared to young animals. Furthermore, we evaluated cell death, astrogliosis, and expression levels of autophagic markers, which all showed to be increased in the aged animal group. Statistically significant alterations in astrocytes number and in mRNA levels of LC3 and p62—both autophagic pathway markers—were observed in the aged group, compared to the young animals. These results show for the first time that the aging process might play a role in SCA2, providing a hint for a probable impact on other neurodegenerative diseases.

Previously, we showed that expanded ataxin-2 expression in the striatum of wild-type C56BL/6 mice leads to the development of neuropathological hallmarks associated with SCA2, being a suitable model for the study of disease, by displaying features such as neuronal death, neuroinflammation, and autophagy impairment [32]. Therefore, we selected this model to study aging’s impact on expanded ataxin-2 expression. A study on *C. elegans* showed that protein aggregates resembling amyloid-like structure are formed in aging, and furthermore the study implied that these aggregates contribute to the aging process [39]. SCA2 typically occurs in adulthood and the onset is correlated with the number of polyglutamine expansions, however, there are reports of different ages of onset in individuals with the same number of polyglutamine expansions. In this line, we hypothesize that mutant ataxin-2 expression in aged animals will exacerbate the neuropathological features, including the number of aggregates and the loss of neuronal markers. In fact, we found that mutant ataxin-2 expression in 18-month-old animals led to an increase in the number of aggregates and in the volume loss of neuronal markers, suggesting that neuronal cell aging intensifies SCA2 disease neuropathology. The SCA2 lentiviral mouse model displayed neuronal death. We found increased neuronal death in the aged animals, suggesting that mutant ataxin-2 expression and aging aggravated cellular injury and death. Astrocytes play a role in the inflammatory response in the central nervous system, and the presence of aggregates was reported in astrocytes of patients with neurodegenerative diseases, hereby disrupting normal astrocyte function [41]. In line with previous results, we found more activated astrocytes in the aged animal group, as compared with the younger animals. Lastly, we evaluated mRNA alterations in specific aging hallmarks in the experimental groups, namely dysregulated proteostasis, mitochondria function, and interneural communication. Previously, we found alterations in the SCA2 lentiviral mouse model concerning autophagy markers [32]. Accordingly, we found significant alterations in LC3 and p62 mRNA levels in the aged group, as compared to younger animals. We did not observe alterations in mRNA levels of genes associated with mitochondrial function or with inter-neuronal communication. These results are in line with the AGEMAP—Atlas of Gene Expression in Mouse Aging Project—which analyzed gene expression alterations in 16 mouse tissues over time [42]. The brain regions analyzed were the cerebellum, cerebrum, hippocampus, and striatum. None of the 8932 genes analyzed were altered in the striata, however in the cerebellum, 17 genes were age-regulated. This might explain why we did not observe alterations in gene expression of genes associated with several aging hallmarks in the striatum.

Our study was conducted in the striatal region of the brain, however, the cerebellum is the most affected brain region in spinocerebellar ataxias, and in SCA2, especially Purkinje cells [43]. Several studies of SCA2 patients reported neurodegeneration of other brain regions, including the striatum [43,44,45], therefore highlighting the importance of these results for future studies evaluating aging’s impact on the cerebellum. This study showed for the first time that the expression of mutant ataxin-2 in different life stages leads to different neuropathological outcomes, being more severe when expressed in later life stages. In addition to the neuropathological hallmarks, we also evaluated cell death markers and astrogliosis, which were increased in the older animal groups injected with mutant ataxin-2. This study shows that aging does impact SCA2 disease progression and severity, suggesting that aging might be an important player in disease onset. As SCA2 disease belongs to the group of polyglutamine disorders, these results might provide a hint for aging’s impact on other polyglutamine and neurodegenerative diseases.

## 4. Materials and Methods

### 4.1. Striatal Lentiviral SCA2 Mouse Model

Three-month-old (*n* = 12) and eighteen-month-old (*n* = 6) C57BL/6 mice were anesthetized with a mixture of medetomidine (0.75 mg/kg, DOMTOR^®^ Esteve, Orionintie, Finland) and ketamine (75 mg/kg, Nimatek, Dechra, Handelsweg, Netherlands) and stereotaxically injected in the striatum, with lentiviral vectors encoding for human ataxin-2 with 23 CAG (WT) repetitions in the left hemisphere, and human ataxin-2 with 82 CAG repetitions (MUTAtxn2) in the right hemisphere, as previously described [32]. The animals were housed at the animal facility of the Algarve Biomedical Center Research Institute of the University of Algarve, with *ad libitum* access to food and water. The environment was temperature-, humidity-, and light-controlled, with 12 h light–12 h dark cycles. The animal experiments were carried out in accordance with the EU directive 2010/63/EU for care and use of laboratory animals. The researchers received adequate training (FELASA-certified course) and certification to perform the experiments from Portuguese authorities (Direção-Geral de Alimentação e Veterinária).

### 4.2. Mouse Brain Tissue Processing

Twelve weeks after the injection, the animals were anesthetized with a lethal mixture of medetomidine (0.75 mg/kg, DOMTOR^®^ Esteve, Orionintie, Finland) and ketamine (75 mg/kg, Nimatek, Dechra, Handelsweg, Netherlands) and sacrificed. All animals selected for immunohistochemical assays were sacrificed by cardiac perfusion with 4% paraformaldehyde (PFA) solution (Sigma Aldrich, Steinheim, Germany). Upon removal, the brains were fixated for 24 h in 4% PFA solution, dehydrated in 20% saccharose solution for 48 h, and cryopreserved at −80 °C degrees. Coronal brain slices of 20 μm were obtained using a cryostat (Cryostar NX50, ThermoFisher Scientific, Walldorf Germany) and were preserved free-floating in 0.05% sodium azide solution at 4 °C. For RT-qPCR analysis, the animals were sacrificed by cervical dislocation. Upon brain removal, we collected a striatal punch using a Harris Core pen with a 2.5 mm diameter (Ted Pella Inc., Redding, CA, USA) and stored at −80 °C until subsequent processing.

### 4.3. Cresyl Violet Staining

Brain sections were mounted on microscopy slides, dehydrated in ethanol and xylene solutions, and stained with cresyl violet differentiated in acetate buffer, pH 3.8–4 (2.72% sodium acetate and 1.2% acetic acid, 1:4 *v*/*v*). Following a second re-dehydration, the sections were mounted with Eukitt^®^ (O. Kindler GmbH & CO, Freiburg, Germany).

### 4.4. Immunohistochemical Procedures

Immunohistochemistry protocols are described elsewhere [46,47]. Briefly, for brightfield microscopy, brain sections were incubated in phenylhydrazine solution (50 mg/mL, Sigma-Aldrich, Saint Louis, MO, USA) for 30 min at 37 °C and washed at room temperature (RT). Sections were blocked (10% Normal goat serum (NGS, Gibco, Penrose, New Zealand) +0.1% triton (Triton X-100)) for 1 h, at RT, and incubated overnight with primary antibody diluted in blocking solution (Table 1), at 4 °C. Then, they were incubated in the secondary antibody for 2 h at RT, followed by a signal-amplifying step using the solution Vectastain ABC kit for 40 min at RT, and by 3,3′-diaminobenzidine substrate (both from Vector Laboratories, Burlingame, CA, USA). Sections were assembled, dehydrated in increasing degree ethanol solutions (75%, 96%, and 100%) and xylene, and cover-slipped using Eukitt^®^ (O. Kindler GmbH & CO, Freiburg, Germany). For fluorescence-labeling procedures, sections were blocked, and probed as described above, using primary (Table 1) and secondary Alexa Fluor-conjugated antibodies (Invitrogen, Eugene, OR, USA). Sections were assembled and cover-slipped using Floromount-G™ with DAPI. Images were acquired with 10× (EC Plan-Neofluar 10×/0.30 Ph1), 20× (EC Plan-Neofluar 20×/0.50 Ph2 M27), and 40× (EC Plan-Neofluar 40×/0.75 Ph2 M27) objectives in a Zeiss Axio Imager Z2 and Axio Scan.Z1 motorized plate microscopes.

### 4.5. Immunohistochemical Quantitative Analysis

Aggregates’ number and DARPP-32 volume loss were quantified using ZEN lite software (Zeiss, Turnstrasse, Germany) by analyzing 18 coronal sections representative of the rostrocaudal striatum. For the astrocyte quantification, three serial sections were selected, representative of the striatal most central location. The aggregates and astrocytes were manually counted in all animals, and the result was multiplied by 8—the distance between serial sections—allowing the quantification of the total number in the striatum. The area of DARPP-32 depletion was manually measured for all animals, and the volume was calculated according to the formula: Volume=d×(a1+a2+a3), where d represents the distance between serial sections (200 μm) and a1 + a2 + a3 are the measured areas for each section. The cleaved caspase-3-positive cells were counted in six sections for each animal, spanning the transduced area of the striatum. The values were plotted as the mean number of cleaved caspase-3-positive cells per area and normalized by the basal values observed in the control hemisphere (expressing WTAtxn2). All measurements were performed in a blind manner.

### 4.6. Real-Time Quantitative PCR

Total RNA extraction was performed using Trizol (Invitrogen, Carlsbad, CA, USA) dissociation and chloroform separation, followed by the NZY Total RNA Isolation kit (Nzytech, Lisboa, Portugal), according to manufacturer instructions. RNA quantification and purity were determined using NanoDrop™ 2000 (Thermo Scientific, Wilmington, De, USA). cDNA synthesis was prepared according to the iScript cDNA kit (Bio-Rad, Hercules, CA, USA) protocol, in a C1000 Touch™ Thermal Cycler(Bio-Rad, Hercules, CA, USA). RT-qPCR was performed using SsoAdvanced™ SYBR^®^ Green Supermix (Bio-Rad, Hercules, CA, USA) using the primers detailed in Table 2 in a QuantStudio 1 Real-Time PCR System ThermoFisher (Life Technologies, Marsiling, Singapore). The amplification values were determined using target mRNA expression levels relative to housekeeping genes’ mRNA. Results obtained from the MUTAtxn2 group were normalized with the respective control group injected with WTAtxn2. 

### 4.7. Statistical Analysis

Statistical analysis was performed using paired and unpaired *t*-tests (two-tailed or with Welch’s correction) and one-way ANOVA, using the GraphPad software (La Jolla, CA, USA). Statistical outliers were determined using Grubbs’ test. Results are expressed as mean ± SEM and significant thresholds were set at * *p* < 0.05, ** *p* < 0.01, *** *p* < 0.001, and **** *p* < 0.0001.

## 5. Conclusions

Altogether, our results showed that aging aggravates some neuropathological features of SCA2, namely by increasing the number of aggregates, increasing the loss of neuronal markers, and increasing neurodegeneration. This increase in neuropathological features was observed in aged animals, compared to young mice, both subjected to the expression of mutant ataxin-2.

Due to the importance of the cerebellum in this disease, future studies should focus on the impact of aging in this brain region, as well as in other markers of aging. Additionally, it is essential for further investigation to characterize the exact mechanism underlying the age increase of neuropathological features. Nevertheless, the results presented in this study open a new avenue for the role of aging in SCA2 and in other polyglutamine diseases.

## Figures and Tables

**Table 1:** Primary and secondary antibodies used in immunohistochemistry (IHC).

**Primary Ab**	**Brand**	**Ref**	**IHC**
mouse anti-ataxin-2	BD Biosciences	611378	1:1000
rabbit anti-DARPP-32	Merk Millipore	AB10518	1:1000
mouse anti-GFAP	Biolegend	644702	1:1000
rabbit anti-cleaved caspase-3 (Asp175)	Cell Signaling	9661	1:1000
**Secondary Ab**			
Alexa anti-mouse 488	Invitrogen	A11001	1:200
Alexa anti-mouse 594	Invitrogen	A11005	1:200
Alexa anti-rabbit 594	Invitrogen	A11012	1:200
Biotinylated anti-Mouse	Vector Laboratories	BA-9200	1:200
Biotinylated anti-Rabbit	Vector Laboratories	BA-1000	1:200

Ab—antibody.

**Table 2:** RT-qPCRs primers list.

Primers	Brand	Fw Sequence	Rv Sequence	Dilution
HPRT	Qiagen	No sequence available from the company	No sequence available from the company	1:10/1:20
ATXN2	Qiagen	No sequence available from the company	No sequence available from the company	1:10
GAPDH	Invitrogen	TTTACTGGCAACATCAACAG	GAATTTCTTAAACGGGAGGC	1:10
LC-3b	Invitrogen	GACGGCTTCCTGTACATGGTTT	TGGAGTCTTACACAGCCATTGC	1:20
Beclin-1	Invitrogen	TTTTCTGGACTGTGTGCAGC	GCTTTTGTCCACTGCTCCTC	1:20
p62	Invitrogen	ATGCTGTCCATGGGTTTCTC	GGTGGAGGGTGCTTTGAATA	1:20
PGC-1α	Invitrogen	AAACTTGCTAGCGGTCCTCA	TGGCTGGTGCCAGTAAGAG	1:10
TFAM	Invitrogen	CCTTCGATTTTCCACAGAACA	GCTCACAGCTTCTTTGTATGCTT	1:10
VGLUT2	Invitrogen	TGCTACCTCACAGGAGAATGGA	GCGCACCTTCTTGCACAAAT	1:10
PSD95	Invitrogen	GACGCCAGCGACGAAGAG	CTCGACCCGCCGTTTG	1:10
mTOR	Invitrogen	TCCTGCGCAAGATGCTCATC	TGTGCTCCAGCTCTGTCAGGA	1:10

## Data Availability

The datasets used and/or analyzed during the current study are available from the corresponding author upon reasonable request.
